# A 21st Century Perspective of Poliovirus Replication

**DOI:** 10.1371/journal.ppat.1004825

**Published:** 2015-06-04

**Authors:** Nicolas Lévêque, Bert L. Semler

**Affiliations:** 1 Clinical and Molecular Virology Unit (EA-4684 CardioVir), School of Medicine, University of Reims Champagne-Ardenne, Reims, France; 2 Center for Virus Research, Department of Microbiology and Molecular Genetics, School of Medicine, University of California, Irvine, Irvine, California, United States of America; University of Michigan Medical School, UNITED STATES

## Why Poliovirus Replication Has Been Studied for More Than 50 Years

**Fig 1:**
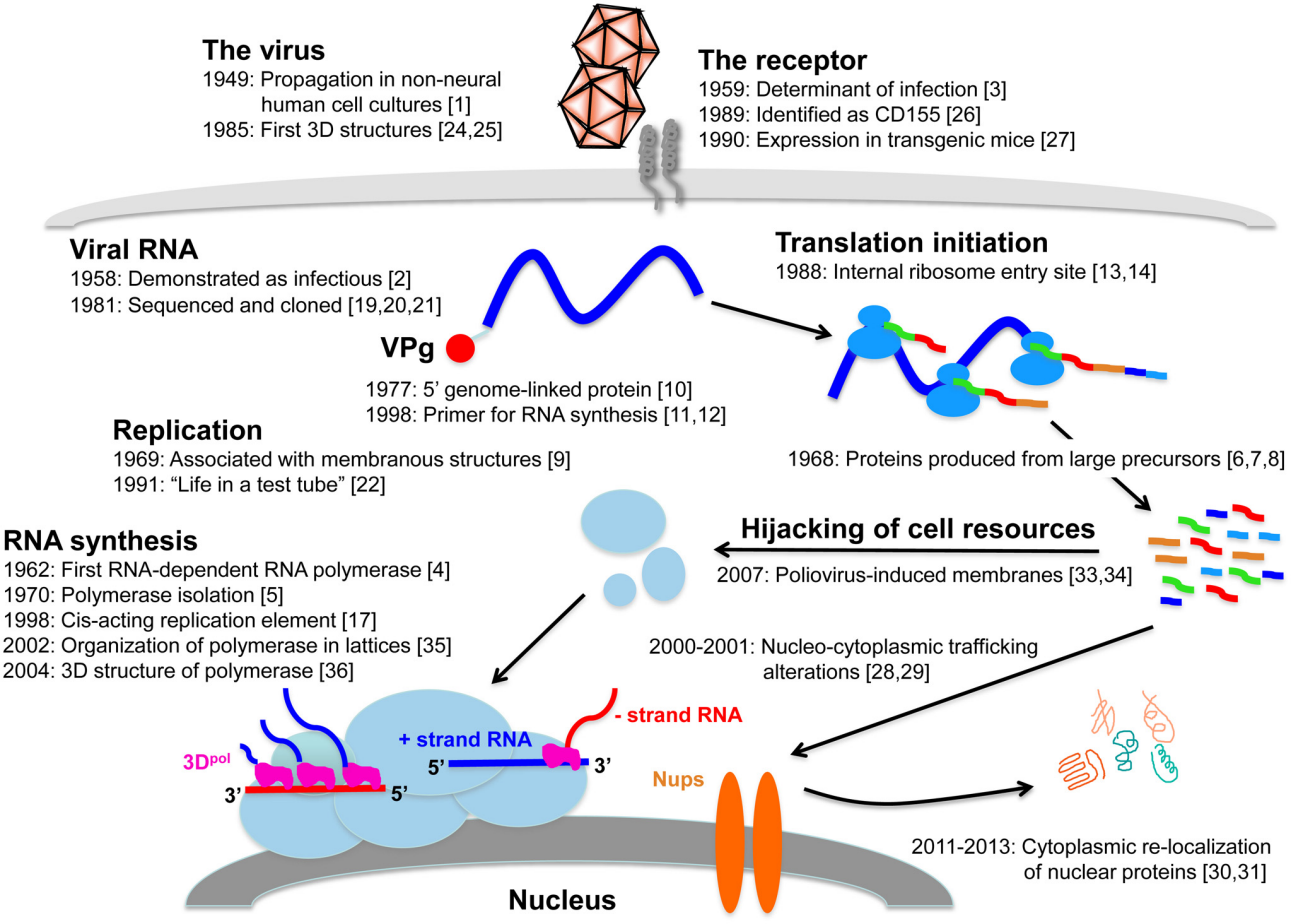
Replication cycle of poliovirus, annotated with references to key findings during the past 65 years. Abbreviations and symbols: 3D, three-dimensional; receptor proteins (grey cylinders spanning plasma membrane); RNA, ribonucleic acid; VPg, viral protein, genome-linked (red oval); ribosomes (medium blue oval shapes bound to viral RNA); + strand RNA, positive-sense genomic RNA (blue horizontal or wavy line);—strand RNA, negative-sense antigenomic RNA (red horizontal or wavy line); 3D^pol^, viral RNA-dependent RNA polymerase (pink shapes); membranous vesicles (light blue oval shapes); Nups, nucleoporins (orange, oblong oval shapes).

Poliovirus is the etiologic agent of poliomyelitis, an acute flaccid paralysis affecting 1%–2% of infected patients and, on rare occasions, causing death by paralyzing muscles that control the throat or breathing. A striking feature of infection is lifelong disabilities that may affect survivors of the acute disease. Transmitted by the fecal—oral and oral—oral route, this virus (three serotypes) was one of the most feared pathogens in industrialized countries during the 20th century affecting hundreds of thousands of children every year, via outbreaks during warm summer months. Although there are highly effective vaccines to control poliomyelitis, it remains endemic in a few countries, from which spread and outbreaks continue to occur throughout the world. Since its discovery in 1908, poliovirus has been intensively studied to better understand and control this formidable pathogen. The history of poliovirus is not, however, limited to the fight against the disease. Poliovirus replication studies also have played important roles in the development of modern virology since poliovirologists and, more generally, picornavirologists have been pioneers in many domains of molecular virology. Poliovirus was, for example, the first animal RNA virus to have its complete genome sequence determined, the first RNA animal virus for which an infectious clone was constructed, and, along with the related rhinovirus, the first human virus that had its three-dimensional structure solved by X-ray crystallography. Indeed, the history of over half a century of poliovirus replication studies is marked by major discoveries, many of which are summarized here and illustrated in Fig 1.

## 1950–1970: The Early Years

In 1949, John Enders, Thomas Weller, and Frederick Robbins performed a landmark study showing that poliovirus could be propagated in cultured, non-neural human cells that did not correspond to the tissues infected during the disease [1]. Not only did these Nobel Prize-winning studies pave the way for the development of highly effective vaccines against poliovirus, but they also opened the door for virologists to study the molecular mechanisms of poliovirus replication in cultured cells that were much more readily manipulated than neural tissue. Isolated poliovirus genomic RNA was then shown to be infectious for susceptible HeLa cells in monolayers, demonstrating that the viral genome itself is the carrier of the biological activity responsible for infection [2]. John Holland and coworkers then reproduced this experiment with normally nonsusceptible cells, demonstrating that the block to poliovirus growth in nonpermissive, nonprimate cells was due to the absence of specific receptors, defining cell determinants of poliovirus infection [3].

The first evidence for an RNA-dependent RNA polymerase used by the virus for RNA genome replication was reported in 1962 by Baltimore and Franklin [4]. Until then, it was believed that the genome of RNA viruses was replicated in the cell nucleus by a cellular DNA-dependent RNA polymerase. Studies on another picornavirus, mengovirus, revealed the existence of an actinomycin D-resistant replication activity in the cytoplasm of infected cells that was later also identified in poliovirus-infected cells [4]. This virus-specific enzyme was isolated eight years later [5].

In the late 1960s, Summers and Maizel [6], and others [7,8], showed that the genomic RNA of poliovirus is translated to produce very large polypeptides that are then specifically cleaved into smaller functional proteins. This discovery was the first demonstration that picornavirus proteins were produced from large precursors proteolytically processed in infected cells [6]. At the same time, cellular fractionation studies revealed that poliovirus RNAs are synthesized in replication complexes bound to distinct membranous structures in the cytoplasm of infected cells [9].

## 1970–2000: The Late 20th Century

This period corresponds to several major advances in understanding the mechanisms of translation and replication of poliovirus genomic RNA. In 1977, a small protein of 22 amino acids, called VPg, was discovered to be covalently linked to the 5' end of poliovirus RNA [10]. Its absence when the virus genome is translated on polysomes indicated that VPg is removed prior to or during translation, whereas its presence at the 5’ end of the newly synthesized positive- and negative-strand RNAs suggested that VPg might be involved in the initiation of RNA synthesis [11]. This hypothesis was confirmed later by Paul et al., who demonstrated that VPg linked by the poliovirus RNA polymerase 3D^pol^ to the 5'-terminal uridylic acid of the virus genome is the key player in protein-primed initiation of poliovirus RNA synthesis [12]. Experiments with dicistronic vectors subsequently led to the discovery of a novel mechanism of initiation of translation through ribosome binding to RNA secondary structures within the long 5' nontranslated region of the virus genome. This internal ribosome entry site (or IRES) allows recognition by the host translation apparatus in the absence of a 5’ cap structure on viral mRNAs [13,14]. Since infection by poliovirus and many other picornaviruses leads to the shut off of cap-dependent translation [15,16], the IRES allows the virus to effectively compete for the cellular translation machinery via cap-independent mechanisms. Finally, a cis-acting replication element (or cre) was identified in picornavirus genomic RNAs. Cre sequences are RNA stem-loop structures almost exclusively located within the coding region, and they are required for viral RNA replication. These elements bind viral proteins involved in RNA replication complex formation, allowing specific recognition of viral RNAs in the cytoplasm of infected cells among a myriad of poly(A)-containing host cellular mRNAs. Cre sequences promote uridylylation of VPg, the primer for initiation of viral RNA synthesis, by 3D^pol^. Their functions appear to be strand-specific, since the cre is required for positive-strand RNA synthesis but may not be essential for negative-strand RNA synthesis. A cre was first found in the capsid coding region of human rhinovirus (HRV) RNA and later in the 2C coding region of poliovirus RNA [17,18]. Since then, similar types of internal recognition elements have been detected for other positive-strand RNA viruses.

The complete sequence of poliovirus genomic RNA was reported in 1981 by two different groups [19,20]. Poliovirus RNA was shown to be more than 7,400 nucleotides long, polyadenylated at the 3' terminus, and composed of an open reading frame (ORF) of 2,207 consecutive triplets, spanning over 89% of the total nucleotide sequence. This single ORF encodes the viral polyprotein that is ultimately cleaved into more than 15 intermediate and mature viral polypeptides. Poliovirus was also the first RNA animal virus for which an infectious, cloned complementary DNA copy of the RNA genome was constructed. Transfection of this clone into mammalian cells produced infectious poliovirus and, via genetic manipulation, led to new insights about the functions of viral proteins and RNA sequences and their roles in the picornavirus intracellular replication cycle [21].

Transcribed viral RNA from a plasmid-derived cDNA was also used in vitro for the de novo synthesis of infectious poliovirus, a first for an animal RNA virus. Published in 1991 by Eckard Wimmer’s group, "Life in a Test Tube" resulted from the addition of viral RNA to a crude cytoplasmic extract of uninfected HeLa cells. These extracts contain all essential elements for poliovirus replication, including cytoplasmic membranes and components required for virion assembly [22]. Implementation of this system allowed the use of “in vitro genetics” with defined templates and fractionated extracts to understand many aspects of the virus life cycle, especially the mechanism of initiation of RNA synthesis to yield VPg-linked progeny RNAs [23].

Based upon X-ray crystallography studies, poliovirus and its relative, HRV, were the first animal viruses for which the three-dimensional virus structure was solved in 1985 by the groups of Hogle and Rossmann [24,25]. And in 1989, Racaniello and coworkers identified CD155, a member of the immunoglobulin superfamily, as the poliovirus receptor [26]. This finding was followed by the generation of mice carrying CD155 as a transgene, allowing studies of poliovirus infection and pathogenesis in vivo in a nonprimate model [27].

## 2000–2015: The 21st Century

Numerous studies carried out during this period uncovered the ability of poliovirus to usurp cellular components and structures for its own benefit, reflecting the necessity for this virus with a very limited coding capacity to hijack cell resources during infection. Relocalization of nuclear proteins in the cytoplasm during poliovirus infection was first reported in 2000–2001. Cleavage of nuclear pore complex components such as Nup153 and p62 by the viral 2A proteinase led to the accumulation in the cytoplasm of virus-infected cells of a number of proteins normally present in the nucleus [28,29]. These relocalized proteins often manifest RNA binding capabilities and function in host cell RNA metabolism steps. For example, the nuclear protein SRp20, a cellular splicing factor, is relocalized to the cytoplasm of poliovirus-infected human cells. It was identified as an important IRES trans-acting factor (ITAF) for poliovirus translation [30]. Another cellular protein that is redistributed in the cytoplasm during poliovirus infection is 5'-tyrosyl-DNA phosphodiesterase-2 (TDP2), a DNA repair enzyme identified as the source of VPg unlinkase activity that cleaves the protein-RNA covalent linkage of VPg at the 5’ end of virion RNA [31,32]. VPg unlinkase/TDP2 activity may be essential to provide a balance between the translation, replication, and packaging functions of viral RNA.

Poliovirus replication sites on cellular membranes (first described in 1969) were also shown to be the result of viral hijacking of components of cellular membrane metabolic pathways, leading to intracellular membrane remodeling and generation of specialized sites distinct in protein and lipid composition from that of the host cell. Belov, Altan-Bonnet, and colleagues demonstrated that viral proteins promoted membrane-binding of members of the Arf family of small guanosine triphosphatases (GTPases), leading to the formation of phosphatidylinositol-4-phosphate lipid-enriched organelles that are essential for poliovirus RNA replication [33, 34]. Indeed, these poliovirus-induced membranes contain viral RNA-dependent RNA polymerases organized in lattices of hundreds of molecules observed for the first time in 2002 by electron microscopy [35]. The two-dimensional planar and tubular oligomeric arrays of poliovirus polymerase display cooperative RNA binding and elongation. Understanding the nature of polymerase interface domains, polymerase activation, and template RNA/rNTP interactions has been greatly facilitated by the complete structure of the poliovirus RNA polymerase, which was reported in 2004 [36]. Combining our knowledge of polymerase structure, function, and genetics has also revealed novel insights into how poliovirus polymerase fidelity contributes to the quasi-species nature of RNA virus populations [37,38].

## Why Poliovirus Replication Must Still Be Studied

Despite a dramatic reduction in the number of cases of poliomyelitis (350,000 cases in 1988 versus 416 cases in 2013), as well as of polio endemic countries (125 countries in 1988 versus 3 countries in 2014), poliovirus is not yet eradicated from our planet and is still the target of a massive worldwide vaccination campaign (http://www.polioeradication.org/). Moreover, vaccine-associated paralytic poliomyelitis resulting from the use of a live, attenuated vaccine complicates any poliovirus endgame strategy [39]. Finally, enteroviruses other than poliovirus have emerged as serious threats to public health. These include enterovirus 71, responsible in infants and young children for hand, foot, and mouth disease with the potential for severe central nervous system complications, and enterovirus D68, detected in children hospitalized with severe lower respiratory symptoms and asthma. In this context, there is an absolute necessity to identify potential new targets for novel antienteroviral drugs and develop new, safe, and effective vaccines. Given the plethora of cellular, molecular, and genetic tools produced during the study of poliovirus replication for more than half a century, it is paramount to continue to use these invaluable research tools to expand our knowledge of the replication and virulence determinants of emerging enteroviruses. In addition, there is still much to be learned about the pathogenic mechanisms of enteroviruses as well as the mechanistic details of poliovirus replication, particularly how the virus weaves the functions of host cell activities into a complex web of intracellular events in its reproductive cycle. Such information may be required to implement the final push that could ultimately result in the eradication of this legendary human pathogen.

## References

[1] Enders J. F., Weller T. H., and Robbins F. C.. 1949 Cultivation of the Lansing strain of poliomyelitis virus in cultures of various human embryonic tissues. Science 109:85–87. 17794160 10.1126/science.109.2822.85

[2] Alexander H. E., Koch G., Mountain I. M., and Van Damme O.. 1958 Infectivity of ribonucleic acid from poliovirus in human cell monolayers. J. Exp. Med. 108:493–506. 13575680 10.1084/jem.108.4.493 PMC2136898

[3] Holland J. J., M. L. C., and Syverton J. T.. 1959 Mammalian cell-virus relationship. III. Poliovirus production by non-primate cells exposed to poliovirus ribonucleic acid. Proc. Soc. Exp. Biol. Med. 100:843–845. 13645739 10.3181/00379727-100-24798

[4] Baltimore D., and Franklin R. M.. 1962 Preliminary data on a virus-specific enzyme system responsible for the synthesis of viral RNA. Biochem. Biophys. Res. Commun. 9:388–392. 13966258 10.1016/0006-291x(62)90021-9

[5] Ehrenfeld E., Maizel J. V., and Summers D. F.. 1970 Soluble RNA polymerase complex from poliovirus-infected HeLa cells. Virology 40:840–846. 4317360 10.1016/0042-6822(70)90129-7

[6] Summers D. F., and Maizel J. V.. 1968 Evidence for large precursor proteins in poliovirus synthesis. Proc. Natl. Acad. Sci. U.S.A. 59:966–971. 4296044 10.1073/pnas.59.3.966 PMC224791

[7] Holland J. J., and Kiehn E. D.. 1968 Specific cleavage of viral proteins as steps in the synthesis and maturation of enteroviruses. Proc. Natl. Acad. Sci. U.S.A. 60:1015–1022. 4299264 10.1073/pnas.60.3.1015 PMC225154

[8] Jacobson M. F., and Baltimore D.. 1968 Polypeptide cleavages in the formation of poliovirus proteins. Proc. Natl. Acad. Sci. U.S.A. 61:77–84. 4301595 10.1073/pnas.61.1.77 PMC285907

[9] Caliguiri L. A., and Tamm I.. 1969 Membranous structures associated with translation and transcription of poliovirus RNA. Science 166:885–886. 4310189 10.1126/science.166.3907.885

[10] Lee Y. F., Nomoto A., Detjen B. M., and Wimmer E.. 1977 A protein covalently linked to poliovirus genome RNA. Proc. Natl. Acad. Sci. U.S.A. 74:59–63. 189316 10.1073/pnas.74.1.59 PMC393196

[11] Nomoto A., Detjen B., Pozzatti R., and Wimmer E.. 1977 The location of the polio genome protein in viral RNAs and its implication for RNA synthesis. Nature 268:208–213. 196204 10.1038/268208a0

[12] Paul A. V., van Boom J. H., Filippov D., and Wimmer E.. 1998 Protein-primed RNA synthesis by purified poliovirus RNA polymerase. Nature 393:280–284. 9607767 10.1038/30529

[13] Pelletier J., and Sonenberg N.. 1988 Internal initiation of translation of eukaryotic mRNA directed by a sequence derived from poliovirus RNA. Nature 334:320–325. 2839775 10.1038/334320a0

[14] Jang S. K., Krausslich H. G., Nicklin M. J., Duke G. M., Palmenberg A. C., and Wimmer E.. 1988 A segment of the 5' nontranslated region of encephalomyocarditis virus RNA directs internal entry of ribosomes during in vitro translation. J. Virol. 62:2636–2643. 2839690 10.1128/jvi.62.8.2636-2643.1988 PMC253694

[15] Fernandez-Munoz R., and Darnell J. E.. 1976 Structural difference between the 5' termini of viral and cellular mRNA in poliovirus-infected cells: possible basis for the inhibition of host protein synthesis. J. Virol. 18:719–726. 178904 10.1128/jvi.18.2.719-726.1976 PMC515600

[16] Ehrenfeld E., and Lund H.. 1977 Untranslated vesicular stomatitis virus messenger RNA after poliovirus infection. Virology 80:297–308. 196392 10.1016/s0042-6822(77)80006-8

[17] McKnight K. L., and Lemon S. M.. 1998 The rhinovirus type 14 genome contains an internally located RNA structure that is required for viral replication. RNA. 4:1569–1584. 9848654 10.1017/s1355838298981006 PMC1369726

[18] Goodfellow I., Chaudhry Y., Richardson A., Meredith J., Almond J. W., Barclay W., and Evans D. J.. 2000 Identification of a cis-acting replication element within the poliovirus coding region. J. Virol. 74:4590–4600. 10775595 10.1128/jvi.74.10.4590-4600.2000 PMC111979

[19] Kitamura N., Semler B. L., Rothberg P. G., Larsen G. R., Adler C. J., Dorner A. J., Emini E. A., Hanecak R., Lee J. J., van der Werf S., Anderson C. W., and Wimmer E.. 1981 Primary structure, gene organization and polypeptide expression of poliovirus RNA. Nature 291:547–553. 6264310 10.1038/291547a0

[20] Racaniello V. R., and Baltimore D.. 1981 Molecular cloning of poliovirus cDNA and determination of the complete nucleotide sequence of the viral genome. Proc. Natl. Acad. Sci. U.S.A. 78:4887–4891. 6272282 10.1073/pnas.78.8.4887 PMC320284

[21] Racaniello V. R., and Baltimore D.. 1981 Cloned poliovirus complementary DNA is infectious in mammalian cells. Science 214:916–919. 6272391 10.1126/science.6272391

[22] Molla A., Paul A. V., and Wimmer E.. 1991 Cell-free, de novo synthesis of poliovirus. Science 254:1647–1651. 1661029 10.1126/science.1661029

[23] Barton D. J., and Flanegan J. B.. 1993 Coupled translation and replication of poliovirus RNA in vitro: synthesis of functional 3D polymerase and infectious virus. J. Virol. 67:822–831. 8380467 10.1128/jvi.67.2.822-831.1993 PMC237436

[24] Hogle J. M., Chow M., and Filman D. J.. 1985 Three-dimensional structure of poliovirus at 2.9 A resolution. Science 229:1358–1365. 2994218 10.1126/science.2994218

[25] Rossmann M. G., Arnold E., Erickson J. W., Frankenberger E. A., Griffith J. P., Hecht H. J., Johnson J. E., Kamer G., Luo M., Mosser A. G., and et al 1985 Structure of a human common cold virus and functional relationship to other picornaviruses. Nature 317:145–153. 2993920 10.1038/317145a0

[26] Mendelsohn C. L., Wimmer E., and Racaniello V. R.. 1989 Cellular receptor for poliovirus: molecular cloning, nucleotide sequence, and expression of a new member of the immunoglobulin superfamily. Cell 56:855–865. 2538245 10.1016/0092-8674(89)90690-9

[27] Ren R. B., Costantini F., Gorgacz E. J., Lee J. J., and Racaniello V. R.. 1990 Transgenic mice expressing a human poliovirus receptor: a new model for poliomyelitis. Cell 63:353–362. 2170026 10.1016/0092-8674(90)90168-e

[28] Belov G. A., Evstafieva A. G., Rubtsov Y. P., Mikitas O. V., Vartapetian A. B., and Agol V. I.. 2000 Early alteration of nucleocytoplasmic traffic induced by some RNA viruses. Virology 275:244–248. 10998323 10.1006/viro.2000.0427

[29] Gustin K. E., and Sarnow P.. 2001 Effects of poliovirus infection on nucleo-cytoplasmic trafficking and nuclear pore complex composition. EMBO J. 20:240–249. 11226174 10.1093/emboj/20.1.240 PMC140206

[30] Fitzgerald K. D., and Semler B. L.. 2011 Re-localization of cellular protein SRp20 during poliovirus infection: bridging a viral IRES to the host cell translation apparatus. PLoS Pathog. 7:e1002127 10.1371/journal.ppat.1002127 21779168 PMC3136463

[31] Virgen-Slane R., Rozovics J. M., Fitzgerald K. D., Ngo T., Chou W., van der Heden van Noort G. J., Filippov D. V., Gershon P. D., and Semler B. L.. 2012 An RNA virus hijacks an incognito function of a DNA repair enzyme. Proc. Natl. Acad. Sci. U.S.A. 109:14634–14639. 10.1073/pnas.1208096109 22908287 PMC3437895

[32] Ambros V., Pettersson R. F., and Baltimore D.. 1978 An enzymatic activity in uninfected cells that cleaves the linkage between poliovirion RNA and the 5' terminal protein. Cell 15:1439–1446. 215328 10.1016/0092-8674(78)90067-3

[33] Belov G. A., Altan-Bonnet N., Kovtunovych G., Jackson C. L., Lippincott-Schwartz J., and Ehrenfeld E.. 2007 Hijacking components of the cellular secretory pathway for replication of poliovirus RNA. J. Virol. 81:558–567. 17079330 10.1128/JVI.01820-06 PMC1797456

[34] Hsu N. Y., Ilnytska O., Belov G., Santiana M., Chen Y. H., Takvorian P. M., Pau C., van der Schaar H., Kaushik-Basu N., Balla T., Cameron C. E., Ehrenfeld E., van Kuppeveld F. J., and Altan-Bonnet N.. 2010 Viral reorganization of the secretory pathway generates distinct organelles for RNA replication. Cell 141:799–811. 10.1016/j.cell.2010.03.050 20510927 PMC2982146

[35] Lyle J. M., Bullitt E., Bienz K., and Kirkegaard K.. 2002 Visualization and functional analysis of RNA-dependent RNA polymerase lattices. Science 296:2218–2222. 12077417 10.1126/science.1070585

[36] Thompson A. A., and Peersen O. B.. 2004 Structural basis for proteolysis-dependent activation of the poliovirus RNA-dependent RNA polymerase. EMBO J. 23:3462–3471. 15306852 10.1038/sj.emboj.7600357 PMC516629

[37] Pfeiffer J. K., and Kirkegaard K.. 2003 A single mutation in poliovirus RNA-dependent RNA polymerase confers resistance to mutagenic nucleotide analogs via increased fidelity. Proc. Natl. Acad. Sci. U.S.A. 100:7289–7294. 12754380 10.1073/pnas.1232294100 PMC165868

[38] Vignuzzi M., Stone J. K., Arnold J. J., Cameron C. E., and Andino R.. 2006 Quasispecies diversity determines pathogenesis through cooperative interactions in a viral population. Nature 439:344–348. 16327776 10.1038/nature04388 PMC1569948

[39] Orenstein W. A. 2015 Eradicating polio: how the world's pediatricians can help stop this crippling illness forever. Pediatrics 135:196–202. 10.1542/peds.2014-3163 25548328

